# Application of FDM three-dimensional printing technology in the digital manufacture of custom edentulous mandible trays

**DOI:** 10.1038/srep19207

**Published:** 2016-01-14

**Authors:** Hu Chen, Xu Yang, Litong Chen, Yong Wang, Yuchun Sun

**Affiliations:** 1 Center of Digital Dentistry, Faculty of Prosthodontics, Peking University School and Hospital of Stomatology & National Engineering Laboratory for Digital and Material Technology of Stomatology & Research Center of Engineering and Technology for Digital Dentistry of Ministry of Health; 2 Teaching and Research department of Prosthodontics, Peking University School and Hospital of Stomatology.

## Abstract

The objective was to establish and evaluate a method for manufacture of custom trays for edentulous jaws using computer aided design and fused deposition modeling (FDM) technologies. A digital method for design the custom trays for edentulous jaws was established. The tissue surface data of ten standard mandibular edentulous plaster models, which was used to design the digital custom tray in a reverse engineering software, were obtained using a 3D scanner. The designed tray was printed by a 3D FDM printing device. Another ten hand-made custom trays were produced as control. The 3-dimentional surface data of models and custom trays was scanned to evaluate the accuracy of reserved impression space, while the difference between digitally made trays and hand-made trays were analyzed. The digitally made custom trays achieved a good matching with the mandibular model, showing higher accuracy than the hand-made ones. There was no significant difference of the reserved space between different models and its matched digitally made trays. With 3D scanning, CAD and FDM technology, an efficient method of custom tray production was established, which achieved a high reproducibility and accuracy.

In China^1^, nearly 6.8% of people between the ages of 65 and 74 years old, which accounts for around 10 million people, are completely edentulous, and this proportion is expected to increase with increasing life span. At present, the conventional method of restoring edentulous jaws is through the use of complete dentures^2^,^3^,^4^. Thus, the production of complete denture prostheses is also expected to be a large demand.

Achievement of an excellent prognosis for a complete denture treatment requires four key steps^3^,^4^,^5^: impression and cast of the edentulous jaw, the maxillomandibular relationship recording, design and manufacture of the complete denture and clinical try-in. Of these, an accurate impression and cast is the foundation for the retention and stability of the dentures. A working impression should contain three factors: the precise anatomical landmarks of the edentulous jaws, an appropriate extension range and the functional morphology of surrounding tissues.

According to the impression-taking times, there are primary impression method and secondary impression method^3^,^4^,^6^. The primary impression method uses a suitable stock impression tray and impression material to make the final impression. The stock impression tray, however, rarely matches the shape of a patient’s alveolar and dental arch. This technique cannot guarantee an appropriate extension of the denture border or uniform thickness of the impression material and is, therefore, seldom used in patients^7^. The secondary impression method takes a primary impression and a secondary impression. The primary impression is taken by a stock tray with alginate or impression compound, into which the primary gypsum cast is perfused. After filling the undercuts, wax of a certain thickness (usually 2 mm) is baked and paved onto the cast, to allow some space between the tissue surface of the primary cast and the custom impression tray. Next, a chemical curing resin or photo-curable resin is paved onto the surface of the wax at a thickness of 2 mm. Once the moderate extension margin is trimmed, the tray is then cured. This custom tray is then used to make the final impression. Compared to the primary impression method, the secondary impression method allows for production of a tray that fits well and easily makes an accurate impression of the patient’s jaws^8^,^9^. However, this technique takes extra time and materials, and the deformation of wax when paving onto the cast often leads to non-uniform space for the final impression material.

With the development of digital technology, more manual prosthodontic operations can be replaced by digital methods to improve the efficiency of production and the accuracy. In regards to fixed prostheses, from optical impression to computer-aided design (CAD) and computer-aided manufacture (CAM), a relatively mature digital solution is available^10^,^11^,^12^,^13^. However, there are only a few of publications on the digital design and manufacture of complete dentures and the digital solution for edentulous impression taking is still lacking^14^,^15^. This study, therefore, attempted to explore a digital design methodology for production of a custom dental impression tray. Three-dimensional (3D) scanning technology was used to obtain tissue surface data of a primary impression, and CAD technology was used to design the custom tray. Fused deposition modeling (FDM), a kind of 3D printing technology, was ultimately used to produce the tray. Lastly, areas of potential error and difficulty in the process of digital design and production of the custom tray were also analyzed.

## Materials and methods

### Sample preparation and 3D data acquisition

**Figure 1:**
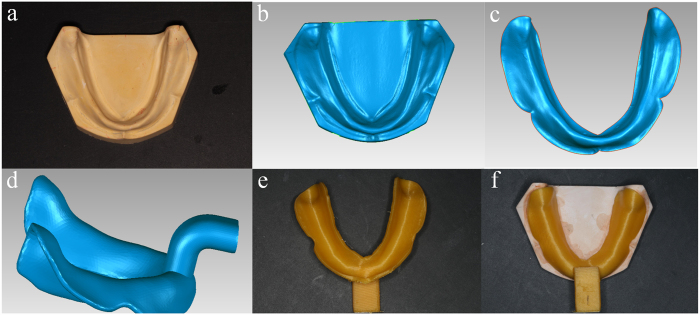
CAD & FDM process for digitally making of mandibular custom tray: (a) the standard mandibular edentulous plaster cast model; (b) the scanned plaster cast model data; (c) the model data was trimmed according to the coverage area of final impression; (d) the designed mandibular custom tray; (e) 3D printed custom tray; (f) the printed tray matched well with the plaster cast model.

Ten standard mandibular edentulous plaster cast models were produced in a same matrix. The tissue surface of the standard plaster cast model was obtained using a 3D model scanner (Activity 880, Smart Optics, Germany), accuracy at 20 μm, and saved in the STL file format (Fig. 1).

### CAD

The scanned data of cast model was imported into a reverse engineering software (Geomagic 2012, Raindrop, USA) where, using an interactive plotted command, the margin of the tray area was extracted and trimmed (Fig. 1). The trimmed surface was then offset along the normal direction by 2 mm, forming an interior surface of the custom tray. To create the solid 3D model of the custom tray body, a 2-mm shell of the interior surface of the tray was drawn out. A handle was also designed and connected to the body of the tray. The designed custom tray data was then saved in the STL file format.

### 3D Printing

The CAD custom tray data was imported into a computer system connected to the FDM printer (Lingtong I, BeijingSHINO, China), and the custom tray was printed layer upon layer at 0.2 mm/layer using polylactic acid (PLA) filament.

### Set up of the control group

**Figure 2:**
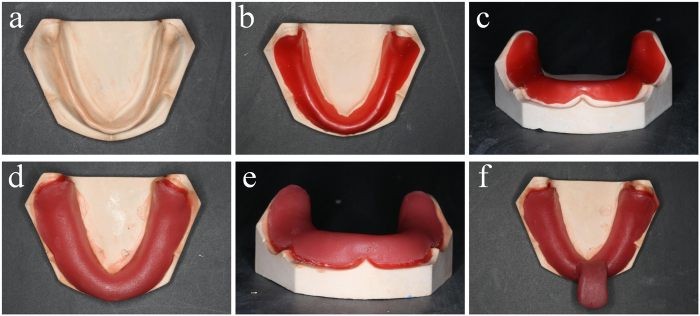
Hand making process of mandibular custom tray: (a) the standard mandibular edentulous plaster cast model; (b) a slice of wax was baked soft to be paved on the cast model (top view); (c) the front view of the paved wax; (d) photo-curable resin is paved onto the surface of the wax, forming a “model-wax-tray” combination (top view); (e) The front view of the “model-wax-tray” combination; (f) A bar was added and the tray finally subjected to light curing for 10 min.

Another ten custom trays were made upon the same mandibular edentulous plaster cast models, using conventional methods (Fig. 2). A 2 mm thick wax was baked to be soft enough, and paved onto the tissue surface of cast model. After the wax cooled down, a slice of photo-curable resin was paved onto the surface of wax and trimmed to appropriate size. Finally a bar was added to the body of custom tray, and the tray was subjected to light curing for 10 min.

### Evaluation of the reserve space of digitally made custom tray

The surface of the FDM printed custom trays were scanned. With registration commands in the Geomagic software, the scanned tray data was registered to its CAD data, so as to put the custom tray virtually in the right place over the model simulating the clinical operation of taking secondary impression. The point-cloud deviation analysis program in another reverse engineering software (Imageware 13.0, EDS, USA) was used to calculate the distance between the tissue surface of custom tray and plaster cast model. Around 500 evenly distributed points were selected, where the distances between FDM printed tray and cast model were measured to evaluate the reserved space for impression material.

### Evaluation of the reserve space of hand-made custom tray

After light curing of the hand-made custom tray, the profile of the model-wax-tray combination was scanned in the 3D model scanner, after which the hand-made tray and model were scanned separately. With registration commands in the Geomagic software, the scanned hand-made tray data and plaster cast model data were registered to the “model-wax-tray” data. The distances between tissue surfaces of tray and model were measured in imageware 13.0 and around 500 points were selected for statistic.

### Statistic methods

The comparison of reserved impression material space between digitally made trays and hand-made trays was performed using independent sample T test. P values equal to or less than 0.05 were considered to be statistically significant. All statistical tests were performed in SPSS version 17.0 (IBM).

### Analysis of error in FDM printing

After registering the tissue surface of FDM printed custom tray to its CAD data in the Geomagic software, the deviation between them was then analyzed in Imageware. The deviation at around 500 evenly distributed points of each tray were selected for statistic, and one way analysis of variance (ANOVA) was used for the analysis.

## Results

As shown in Fig. 1, use of a completely digital methodology allowed for the design and production of a custom tray that fit the plaster model. To better assess the accuracy of the design, consistency of spacing and fit during reproduction were measured.

**Figure 3:**
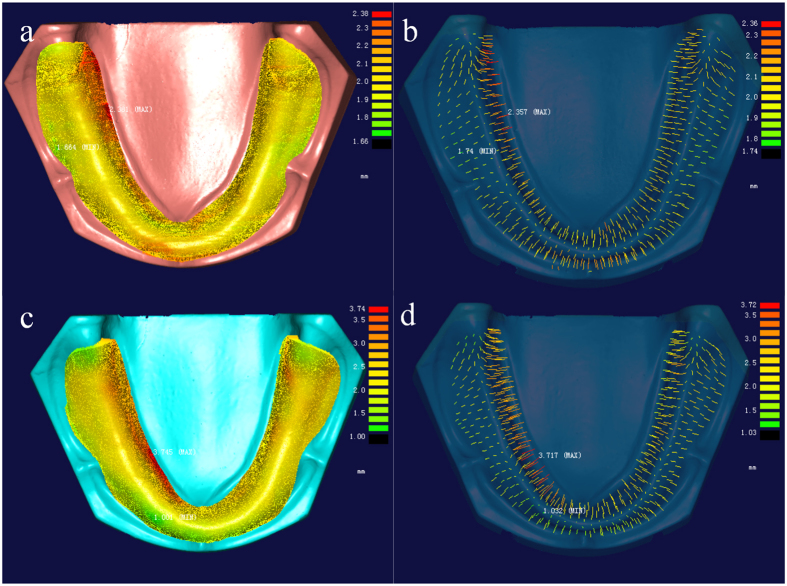
The reserved space between tissue surfaces of model and tray measured in Imageware 13.0: (a) The digitally made custom tray (for model 1); (b) the distances at the selected points of digitally made tray; (c) The hand-made custom tray (for model 1); (d) the distances at the selected points of hand-made tray.

The space between tissue surfaces of cast model and custom tray was expected to be 2.00 mm, while the distance in hand-made tray group showed a lager value than digitally made ones. As the equal variances were not assumed by the levene’s test, the adjusted t-test value was used in Table 1. The mean values and standard deviations of reserved space in hand-made trays (mean: 2.08~2.24 mm; SD: 0.26~0.56 mm) showed significant larger value than the digitally made trays, which had very stable values (mean: 2.01~2.02 mm; SD: 0.09~0.10 mm). The one-way ANOVA of reserved space in digitally made trays shown no significant difference between different models (P = 0.092). Deviation of reserved space occurred in the border area showed lager value than in the central area of the trays despite of different groups (Fig. 3).

**Figure 4:**
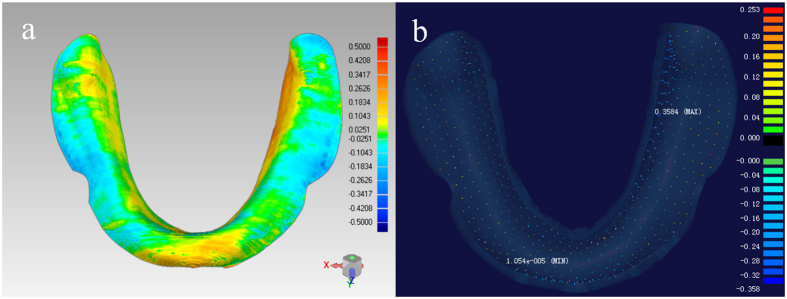
Analysis of FDM printing error of the custom tray for model 1: (a) deviation between tissue surfaces of FDM printed tray and its CAD data (Geomagic 2012); (b) the deviation at selected points (Imageware 13.0).

As shown in Fig. 4, the deviation between the tissue surfaces of the FDM printed tray and its CAD data told the error in the FDM printing step. There also showed a potential of increase of deviation from the central area to the border area, however, the overall deviation was rather low (mean: 0.02~0.03 mm; SD: 0.09~0.10 mm) (Table 2).

## Discussion

Combining 3D data acquisition with CAD/CAM techniques, this study explored a digital methodology for the production of custom trays for edentulous jaw. The CAD process for custom tray design was prepared using Geomagic software, which is a powerful reverse engineering software that has been widely used in the analysis and processing of point-cloud data. In this study, a uniform space of 2 mm between the custom tray and the edentulous jaw model was designed to be reserved for the final impression materials. Specifically, the tray’s tissue surface was generated by moving the primary impression’s surface data at all points along its normal direction by 2 mm using the offset function of the Geomagic software.

Clinically^2^,^4^, the reserved space might present differences when working with different final impression materials. Generally, the 2 mm space is appropriate for alginate, but for impression materials possessing better liquidity, such as silicone rubber, the ideal space should be less. In the Geomagic software, offsets may be customized for both the entire tray, based on the intended impression materials, and for specific regions of the tray. To achieve the effect of selective pressure impression^3^,^4^,^9^, a small offset should be set in the primary and secondary stress-bearing areas, while a larger offset should be set in the area of bony prominences or loose alveolar tissue in need of buffering.

The CAM process of custom tray manufacture can be completed using 3D FDM printing technology^16^,^17^. In this study, a FDM printer was used along with polylactic acid (PLA) filament as the impression material. PLA, also known as polylactide, is a renewable and non-polluting bio-resin extracted from corn and is suitable for medical use^18^,^19^,^20^. After melting the filamentous hot-melt material in the printer, the material is applied to the printer table via a computer-controlled nozzle according to the cross-sectional contour information. The material is rapidly cooled to its final solid form as it is being added. Following the FDM process, the impression is built layer upon layer from the bottom up as the table moves lower following each complete pass of the nozzle.

Compared with the hand-made trays, the reserved space of the digitally made trays showed very small deviation from 2 mm, and no statistically significant difference between the ten different models, achieving a higher accuracy and reproducibility. The relatively high deviation of reserved space in hade-made trays may be caused by the deformation of the baked wax, which didn’t closely fit to the model, resulting in more reserved space.

In the FDM forming process, the major factors affecting the accuracy of the forming include^16^,^21^: the discretization of CAD process, performance of the filament material, exact width of the nozzle, temperature (both temperature of the nozzle and temperature of the forming chamber/table), speed of material extrusion and filling and layer thickness and direction. In this study, the diameter of the 3D FDM printer nozzle is 0.4 mm; the accuracy of its XY axis positioning is 10 microns; and the accuracy of its Z-axis positioning is 5 microns. The layer thickness of the printer can be set from high accuracy (100 microns) to low accuracy (300 microns). The higher the accuracy, the longer it takes to finish the printing process. In this study, medium accuracy (200 microns) was chosen. The printing time for one mandibular custom tray was about 45 min, and the shrinkage of the PLA printing material was 0.3%. If precision of the final tray needs to increase, a higher printing layer precision can be selected.

The ability to design and print a highly accurate custom edentulous impression tray using digital 3D technology is the most important feature of this study. The tissue surface morphology of the digitally designed edentulous tray was matched with the 3D landmarks of the edentulous jaw model, creating a uniform 3D space for the impression materials. The 3D printing method (FDM technology) used in this study has replaced the traditional clinical method consisting of using a pencil to outline the edge of custom trays on plaster casts and wax sheet paving. Although it costs about 50 min for the 3D printing of one mandibular tray, time consumed for the 3D scanning of the primary model and the CAD procedure of the digital tray is only about 10 min (5 min for scanning and 5 min for CAD). As the scanning and printing work can be almost automatically finished by machine, the hand operation time needed is only about 5 min. The overall time of the digital producing procedure will be effectively reduced with the upgrade of the hardware and the development of a specialized custom tray CAD software. The improvements of this methodology will save time in the clinic and also improve the accuracy and overall efficiency of the production process.

Regardless of the technology used to produce a custom tray, when time to make the final impression, it is difficult to determine its exact position and orientation within the patient’s mouth. Furthermore, when the operator lacks experience, placement error will increase. For example, the maldistribution in thickness of the final impression material may lead to the deformation of the alveolar mucosa, thus affecting the accuracy, quality and adaption of the final impression. Further studies are needed on the application of digital design and product technology to improve the precision of edentulous custom tray positioning within the patient’s mouth.

## Additional Information

**How to cite this article**: Chen, H. *et al.* Application of FDM three-dimensional printing technology in the digital manufacture of custom edentulous mandible trays. *Sci. Rep.* **6**, 19207; doi: 10.1038/srep19207 (2016).

## Figures and Tables

**Table 1:** Mean (SD) distance values between cast model and custom trays at the selected points.

Model	RS(mm), Mean ± SD (N)		P	Equality of Variances
	DMTray	HMTray		
1	2.02 ± 0.10 (499)	2.15 ± 0.49 (501)	<0.001	<0.001
2	2.02 ± 0.10 (499)	2.08 ± 0.27 (498)	<0.001	<0.001
3	2.02 ± 0.10 (496)	2.24 ± 0.36 (500)	<0.001	<0.001
4	2.02 ± 0.10 (498)	2.09 ± 0.32 (497)	<0.001	<0.001
5	2.01 ± 0.10 (498)	2.12 ± 0.45 (502)	<0.001	<0.001
6	2.02 ± 0.10 (497)	2.21 ± 0.56 (502)	<0.001	<0.001
7	2.01 ± 0.10 (499)	2.19 ± 0.31 (498)	<0.001	<0.001
8	2.01 ± 0.10 (498)	2.18 ± 0.26 (499)	<0.001	<0.001
9	2.01 ± 0.09 (502)	2.21 ± 0.32 (499)	<0.001	<0.001
10	2.01 ± 0.10 (498)	2.17 ± 0.31 (501)	<0.001	<0.001

RS = Reserved Space; SD = Standard deviation; N = Number of tested points; DMTray = Digitally made tray; HMTray = Hand-made tray; P = P value of independent sample T test; Equality of Variances = Levene’s Test for Equality of Variances.

**Table 2:** Error in FDM printing: deviation between the tissue surfaces of FDM printed tray and CAD data.

Model	Mean (mm)	N	SD (mm)
1	0.02	499	0.10
2	0.02	499	0.10
3	0.02	496	0.10
4	0.03	498	0.10
5	0.02	498	0.10
6	0.03	497	0.10
7	0.02	499	0.10
8	0.02	498	0.10
9	0.02	502	0.09
10	0.02	498	0.10

N = Number of tested points; SD = Standard deviation.

## References

[b1] Hu D. Report on Chinese Oral Health(2012) (social sciences academic press (china), BeiJing, 2011).

[b2] Rahn A. O., Ivanhoe J. R. & Plummer K. D. Textbook of complete dentures, 6th ed. (PMPH-USA, BeiJing, 2009).

[b3] Zarb G. A., Bolender C. L., Eckert S. E., Jacob R. F. & Mericske-Stern R. Prosthodontic treatment for edentulous patients, 13th ed. (Elsevier Mosby, St. Louis, 2013).

[b4] Basker R. M., Davenport J. C. & Thomason J. M. Prosthetic treatment of the edentulous patient (John Wiley & Sons, Chichester, UK, 2011).

[b5] Carlsson G. E. Critical review of some dogmas in prosthodontics. J Prosthodont Res 53, 3 (2009). 19318064 10.1016/j.jpor.2008.08.003

[b6] Rao S., Chowdhary R. & Mahoorkar S. A systematic review of impression technique for conventional complete denture. J Indian Prosthodont Soc 10, 105 (2010). 21629453 10.1007/s13191-010-0020-2 PMC3081259

[b7] Petrie C. S., Walker M. P. & Williams K. A survey of US prosthodontists and dental schools on the current materials and methods for final impressions for complete denture prosthodontics. J Prosthodont 14, 253 (2005). 16359482 10.1111/j.1532-849X.2005.00051.x

[b8] Jo A. *et al.* A randomized controlled trial of the different impression methods for the complete denture fabrication: Patient reported outcomes. J Dent 43, 989 (2015). 26051546 10.1016/j.jdent.2015.05.007

[b9] Hyde T. P., Craddock H. L., Blance A. & Brunton P. A. A cross-over Randomised Controlled Trial of selective pressure impressions for lower complete dentures. J Dent 38, 853 (2010). 20637826 10.1016/j.jdent.2010.07.003

[b10] Cohen B. Digital technology transforms dentistry. Alpha Omegan 107, 7 (2014). 25269217

[b11] Fasbinder D. J. Computerized technology for restorative dentistry. Am J Dent 26, 115 (2013). 23986956

[b12] Andreiotelli M., Kamposiora P. & Papavasiliou G. Digital data management for CAD/CAM technology. An update of current systems. Eur J Prosthodont Restor Dent 21, 9 (2013). 23682504

[b13] Li R. W., Chow T. W. & Matinlinna J. P. Ceramic dental biomaterials and CAD/CAM technology: state of the art. J Prosthodont Res 58, 208 (2014). 25172234 10.1016/j.jpor.2014.07.003

[b14] Bidra A. S., Taylor T. D. & Agar J. R. Computer-aided technology for fabricating complete dentures: systematic review of historical background, current status, and future perspectives. J Prosthet Dent 109, 361 (2013). 23763779 10.1016/S0022-3913(13)60318-2

[b15] Maeda Y., Minoura M., Tsutsumi S., Okada M. & Nokubi T. A CAD/CAM system for removable denture. Part I: Fabrication of complete dentures. Int J Prosthodont 7, 17 (1994). 8179777

[b16] Pennington R. C., Hoekstra N. L. & Newcomer J. L. Significant factors in the dimensional accuracy of fused deposition modelling. Proceedings of the Institution of Mechanical Engineers, Part E: Journal of Process Mechanical Engineering 219, 89 (2005).

[b17] Dudek P. FDM 3D Printing Technology in Manufacturing Composite Elements. Arch Metall Mater 58, 1415 (2013).

[b18] Athanasiou K. A., Niederauer G. G. & Agrawal C. Sterilization, toxicity, biocompatibility and clinical applications of polylactic acid/polyglycolic acid copolymers. Biomaterials 17, 93 (1996). 8624401 10.1016/0142-9612(96)85754-1

[b19] Lunt J. Large-scale production, properties and commercial applications of polylactic acid polymers. Polym Degrad Stabil 59, 145 (1998).

[b20] Pang X., Zhuang X., Tang Z. & Chen X. Polylactic acid (PLA): research, development and industrialization. Biotechnol J 5, 1125 (2010). 21058315 10.1002/biot.201000135

[b21] Anitha R., Arunachalam S. & Radhakrishnan P. Critical parameters influencing the quality of prototypes in fused deposition modelling. J Mater Process Tech 118, 385 (2001).

